# Indigenous microorganisms and benefit sharing

**DOI:** 10.1093/femsyr/foag016

**Published:** 2026-05-05

**Authors:** Katie Lee Riddle, Rogena Sterling, Maui Hudson, Jane Anderson

**Affiliations:** Te Kotahi Research Institute, University of Waikato, Hamilton 3216, New Zealand; Te Kotahi Research Institute, University of Waikato, Hamilton 3216, New Zealand; Te Kotahi Research Institute, University of Waikato, Hamilton 3216, New Zealand; New York University, New York 10012, United States of America

**Keywords:** Indigenous rights, genetic resources, microorganisms, benefit sharing, traditional knowledge, digital sequence information

## Abstract

This article explores the challenges and opportunities associated with benefit sharing and genetic resources in the context of microorganisms, with a particular focus on understanding Indigenous rights and perspectives. It examines traditional uses and understandings of microorganisms by Indigenous communities, evaluates existing international frameworks, and analyses case studies of emerging models of ethical engagement. It argues that benefit sharing mechanisms must be reformed to meaningfully incorporate Indigenous values, interests, and rights, particularly in respect of digital sequence information, and new or emerging biotechnologies.

## Teaser

This article shares Indigenous perspectives, rights, and interests in microbial genetic resources and digital sequence information as well as benefit sharing from their utilization.

## Introduction

The use of microbiology within biotechnology has an established history, and has rapidly accelerated in recent years, particularly through its application in food production and other biotechnologies. It is a growing field of research and development, leading to the development of new and improved products and services at pace (Vitorino and Bessa 2017). Key to this field are Indigenous microorganisms; those which naturally occur within an environment, with strong kinship links to Indigenous peoples, such as the human body, soil, or water. They are often essential to the effective functions of their environments and have the potential to solve particularly difficult problems in life sciences and other fields (Kumar and Gopal 2015). Globally, it is estimated that there are over 1 trillion microbial taxa, and while they are among the most popularly available data and have some of the most phylogenetic and functional diversity, they are often “severely underrepresented or absent” in their species description (Vince et al. 2025).

Indigenous peoples have a deep, profoundly interconnected and symbiotic relationship to their lands, waters, and territories. They have long utilized sophisticated understandings of ecological relationships and fermentation processes in food production, medicine, agriculture, and other cultural practices. Traditional knowledge, developed by Indigenous peoples, particularly that of relevance to microbiology, has garnered the increasing attention of researchers, biotechnology companies, and pharmaceutical firms alike (Rieger et al. 2024). The cause of this increase is largely due to emergence of the bioeconomy as well as the high level of potential for economic benefit or innovation that could be derived from microorganisms and their uses (Bugge et al. 2016).

The ability to sequence, digitize, and manipulate genetic information as part of biodiscovery has transformed how genetic resources are accessed and used, creating new challenges for the traditional legal frameworks designed to protect Indigenous rights and interests. Commercialization of genetic resources and their derivatives have also led to discussions about rights, interests, attribution, access, and benefit sharing when research derives from genetic resources or utilizes traditional knowledge. This is particularly pertinent when Indigenous communities have been excluded from the research process and may miss out on the benefits that result from their contributions to science (Bader et al. 2023).

Digital sequence information derived from microorganisms can now be shared globally without the physical transfer of biological materials, creating the potential risk for circumvention of existing access and benefit sharing mechanisms (Laird et al. 2020). Moreover, the data sharing practices of the open science movement and the commercial reality of many innovation processes, utilize aggregated genetic resources and/or digital sequence information from many different sources, increasing the difficulty of adhering to the Nagoya Protocol (Raposo et al. 2024). This form of avoidance has partially been rectified by the introduction of the United Nations Convention on Biological Diversity (CBD) Digital Sequence Information Multilateral Mechanism and the associated global benefit sharing fund, called the Cali Fund. However, the mechanism only applies to commercial users of digital sequence information and compliance is purely voluntary (CBD 2024).

This paper examines the different perspectives and understandings of research and development with microorganisms, the distinct rights and interests that Indigenous peoples claim in relation to them and analyses the benefit sharing mechanisms, which may be applicable during research and commercialization processes involving microorganisms. This will be predominantly explored through a Māori perspective, drawing on terms and concepts from a Māori worldview. While many Indigenous communities worldwide can share similar values, themes and contexts, the perspectives presented in this paper do not represent the full range of perspectives of all Indigenous peoples globally. Rather, it holds a contextualized lens to a nuanced issue so that key issues may be discussed. It explores the protections afforded by international frameworks, such as those developed by the World Intellectual Property Organization (WIPO) and the CBD and considers potential benefit sharing approaches using the examples provided by both commercial entities Basecamp Research, Variant Bio and a not-for-profit organization, the Fungi Foundation. Together this will illustrate a range of emerging models of equitable engagement with Indigenous communities.

## Indigenous communities and microorganisms

Indigenous communities have developed systems concerning relational ecologies over hundreds of years (Warbrick et al. 2023). Their deep connection to the land and environment has led to an understanding of microorganisms that has been applied in food preservation, medicine, and sustainable practices (Warbrick et al. 2023). Traditional concepts of ecological well-being demonstrate an understanding of the interdependence of flora and fauna (Amazon Watch 2016, Villani et al. 2023), and that different plants of the same species could have stronger medicinal efficacy depending on its specific location and associated unseen but known (microbial) communities. In Aotearoa New Zealand, a te reo Māori (Māori language) term that can be used to understand this is mauri (life essence). It is believed that the microbiome affects the mauri of a taonga species (iconic or culturally significant flora or fauna species) (Maui Hudson et al. 2021). There is only a single generic term for microorganisms in te reo Māori currently used in science; moroiti, but traditional fermentation practices demonstrate the wide practical uses of different microbial processes to preserve foods safely for several plants and seafoods (Whyte et al. 2001).

In the context of microorganisms, the basis for an argument for benefit sharing is complex. Although microorganisms are not visible to the human eye, Indigenous knowledge systems have long recognized their importance and influence. This raises important legal and ethical questions: Should benefit sharing only apply where traditional knowledge is proven, or also where plants come from Indigenous lands with a known history of use? If a genetic resource originates from an Indigenous territory, is that sufficient to trigger benefit sharing obligations? Indigenous frameworks assert that relational custodianship itself is a basis for entitlements, regardless of whether specific traditional knowledge is held about the resource. This also reflects the increasing recognition of Indigenous worldviews where the locations themselves have legal rights and standing (Rapid Transitions Alliance 2018).

## Terminology

Depending on whether the audience is primarily researchers or Indigenous communities, the use of the terms native, Indigenous, and endemic can create misunderstandings, particularly as their uses overlap in the context of both microorganisms and peoples. There is a misconception that a species must be endemic to be culturally significant, but this is not the case. A summary of distinctions between terminology can be found in Table 1.

**Table 1:** Comparison of endemic, native, and indigenous organisms.

Term	Scope	Exclusivity	Legal/cultural context	Example
Endemic	Found naturally in that area	Yes, only found in that area	May have legal protection as a native species	Kawakawa (*P. excelsum*) is endemic to Aotearoa NZ
Native	Found naturally in that area	No, can be found in multiple regions	May have legal protection as a native species	Raupō (*Typha orientalis*) is found in Aotearoa NZ and across the Pacific
Indigenous	Found naturally in that area, and often associated with cultural relationships	May be endemic or native, but is often associated with traditional knowledge	May have legal protection as a native species, and may have cultural protections and usage rights	Tītī (Sooty Shearwater) are endemic to Aotearoa NZ and are culturally significant
Nonindigenous	Brought into the area	No, can be found in multiple regions and/or countries	May have cultural protections	Kūmara (Sweet Potato) are non-Indigenous to Aotearoa NZ and are culturally significant

Native organisms occur “naturally in a particular region without human intervention” (Anastasakis 2021), that is, they have originated from or arrived in an environment naturally and reproduced there over time. Indigenous peoples and nature are often conflated into the term “native,” leading to the compounding of some of the definitional terms seen here.

Indigenous indicates the organisms that are the original inhabitants of a region predating colonization or before significant outside influence. It also infers a stronger cultural and historical context, often linked to self-determination, sovereignty, and ancestral rights. For people, it generally means being part of a distinct community that has maintained cultural ties to their ancestral lands (e.g. Indigenous peoples of Australia, First Nations in Canada). Indigenous is often used as a synonymous term with native organisms, however, it can also include introduced species that have a long history of cultural significance and utility. For example, the kiore (polynesian rat), kurī (polynesian dog), and kūmara (sweet potato) arrived in Aotearoa New Zealand with the early Polynesian voyagers and became staples of Māori society (Waitangi Tribunal 2011). Ko Aotearoa Tēnei, the Waitangi Tribunal report on Cultural Intellectual Property, acknowledges the Indigenous status of both native and culturally significant introduced species under the umbrella term “taonga species.”

Endemic refers to species (plants, animals, or other organisms) that are found exclusively in a limited geographic area and nowhere else naturally (Anastasakis 2021). It is more restrictive than native, as it implies that the species evolved and remains confined to that region. It can occur at various scales, for example, an animal might be endemic to a continent, a country, or even a single island or mountain range. Endemic species only prosper under a certain set of specialized conditions within a specific habitat (Illsley 2018). Aotearoa New Zealand has a high level of endemic biodiversity with an estimated 80 000 species of native animals, plants, and fungi. Examples of endemic species include all frogs and reptiles, more than 90% of insects, ∼80% of vascular plants, a quarter of bird species, and upwards of 40% of marine species (Biosafety Unit 2025). Endemic is only used in the context of species, diseases (Medley and Vassall 2017), and knowledge (Das 2024), but is not used to describe Indigenous Peoples or Local Communities (Illsley).

All endemic species are native to a specific location. However, this also depends on which level of taxa is being compared. The *Piperaceae* family, commonly known as the pepper family, has species with diverse geographic distributions, but some members are endemic to specific regions. For example, kawakawa and kava, while sharing a similar name and belonging to the same plant family, are distinct plants. Kawakawa (*Piper excelsum*) is endemic to Aotearoa New Zealand, while kava (*Piper methysticum*) is native to the South Pacific. Māori named kawakawa, meaning “bitter,” after kava, which they could not cultivate in Aotearoa New Zealand due to the colder climate. Kava is known for its psychoactive and sedative properties, while kawakawa is used medicinally for skin conditions and pain relief. Both kawakawa and kava would be considered Indigenous plants based on natural occurrence as well as cultural significance.

### Non-Indigenous species of significance

Non-Indigenous species of significance recognizes taonga species that Māori have kaitiaki (guardianship) relationships with that are not always considered “Indigenous” to Aotearoa New Zealand. These include species often brought to Aotearoa New Zealand before 1769 on waka migrating from other parts of the Pacific region, i.e. kūmara and kiore (Polynesian rat) (Waitangi Tribunal 2011, pp. 2, 309).

## Examples of common traditional practices that utilized microorganisms

### Food fermentation and preservation

Traditional communities have used fermented foods and drinks for thousands of years (Tamang et al. 2020), enabling survival in challenging environmental conditions. Fermented foods create unique flavors, appearances, and health properties, which have become central to the diet of Indigenous communities (Tamang et al. 2020), and created distinctive flavor profiles and properties that are tied to specific geographic locations and cultural traditions (Paxson 2008). They also offer nutritional benefits and led to the lowering of infectious disease and improved health outcomes (Rangiwai 2021).

### Fermented drinks

A key example of microbial application in traditional knowledge systems is fermented drinks (Worku et al. 2016). In African contexts, beverages like palm wine are produced using mixed cultures of microorganisms, particularly lactic acid bacteria and yeasts (Worku et al. 2016), showcasing deep microbial expertise. Similar practices are found among Māori in Aotearoa New Zealand. Traditional Māori paikaka (homebrews), incorporated native plants such as kūmarahou, kohekohe, and rimu (meaningoftrees 2019). The overripe flowers of the kiekie vine were known to ferment naturally, producing a mildly alcoholic beverage. Kawakawa was also brewed into an aromatic drink by Māori and early missionaries (meaningoftrees).

### Fermented food

While some foods, such as kūmara, could easily be stored in a dry place away from contamination and without elaborate treatment, other food types required fermentation or other food preservation for safe consumption (Whyte et al. 2001). One such example is kānga pirau, a traditional Māori fermentation method in which corn cobs are soaked in running water for several weeks to ferment, producing a pungent and culturally significant food (Asmundson et al. 1994). Tiroi, a preserved combination of mussels and puha (sow thistle), also reflects microbial knowledge, sometimes involving lactic acid fermentation. Other traditionally fermented foods in Aotearoa New Zealand include kina, tītī, and preserved tuna. Additionally, practices like “back-slopping,” in which a portion of an existing ferment is used to cultivate a new batch, illustrate an advanced understanding of microbial selection and cultivation (Tamang et al. 2020).

### Medicinal applications

In medicine, traditional healers have utilized the antimicrobial and healing properties of microorganisms present in plants and natural substances. Māori healers in Aotearoa New Zealand used harakeke, kawakawa, kūmarahou, and mānuka for healing cuts, treating colds, and supporting health (Te Uru Rākau NZ Forest Service 2025). Traditional healers of Indigenous communities in Australia have extensive knowledge in interpretation of symptoms and providing traditional healing treatments, including bush rubs and medicines including those used as antiinflammatory (Australian Indigenous HealthInfoNet 2024). The traditional use of stingless bee honey in the Amazon further illustrates the global diversity of microbial medicinal practices (McCoy 2023).

## Indigenous perspectives of ownership and interests

While Indigenous peoples have recognized rights and interests in relation to genetic resources, the nature of these rights in relation to the genes of microorganisms has yet to be explicitly clarified. Different regimes utilize different rationale for recognizing rights to benefit from different biological and/or environmental resources. In some instances, legal ownership of the resource has been granted [e.g. Ngāi Tahu ownership of pounamu (Aotearoa New Zealand greenstone)], in other instances benefits are achieved through access rights (e.g. mining minerals on tribal lands), and for genetic resources the right to benefit has been linked to associated traditional knowledge. Many Indigenous communities view themselves as stewards or guardians, rather than owners of biological resources, with responsibilities to maintain these resources for future generations. This worldview is grounded in relational ontology, which recognizes humans as part of an interconnected web of relationships with all living beings, including microorganisms (Whyte et al. 2001, Handsley-Davis et al. 2023). There is also a strong emphasis on intergenerational responsibility, underscoring the duty to preserve resources and knowledge for those yet to come, and the principle of reciprocity, which calls for giving back to nature in exchange for what is taken. This relational and ethical framework stands in stark contrast to Western notions of private property and exclusive ownership, posing significant challenges for integrating Indigenous perspectives within existing intellectual property regimes (Handsley-Davis et al. 2023). While Indigenous perspectives on ownership and control of microorganisms differ fundamentally from Western legal frameworks, reflecting distinct cultural, spiritual, and epistemological traditions, there are a wide range of ways to ensure Indigenous rights and interests are not just respected, but result in tangible benefits.

As Bader et al. (2023) notes, while researchers increasingly “seek to understand the complex relationships between humans and their microbiota,” the ethical and relational dimensions of this work have not kept pace with technical advancements. As a result, communities historically marginalized in genomic and biomedical “remain at risk of further exploitation,” and the damage done to relationality, both with Indigenous communities and the microorganisms themselves, continues to reverberate (Bader et al. 2023, p. 1768, Handsley-Davis et al. 2023). A historically significant example illustrating the tensions between traditional knowledge and external research practices arose in 1988, when Māori discovered that the Department of Scientific and Industrial Research had deposited several cultivars of kūmara originally brought to Aotearoa New Zealand by Māori, at a research institution in Japan (Huria 2020, p. 55). These cultivars were no longer available in Aotearoa New Zealand, raising concerns about the ease with which Indigenous flora and fauna could be appropriated by overseas interests without knowledge and/or Māori consent. Māori expressed strong opposition to exclusion from the decision-making process, asserting that both the Government and that the Department of Scientific and Industrial Research had disregarded their rights to tino rangatiratanga (self-determination) and kaitiakitanga over Indigenous biological resources (Huria 2020). This case highlights the ongoing struggle for recognition and protection of Indigenous flora and fauna, taonga species, traditional knowledge, and cultural authority within scientific and legal frameworks, which continue to be pertinent issues today. A contemporary example of the balancing of this tension concerns the genetic sequencing of the Kākāpō in 2019, a taonga species for Ngāi Tahu, to assist species recovery (Guhlin et al. 2023). The project raised questions around governance of the data generated through the project. The samples were obtained using a data sharing agreement that incorporated Indigenous data sovereignty principles and guaranteed that Ngāi Tahu retained kaitiakitanga over the data (Dussex et al. 2021). Future access to the data is controlled, with applicants required to describe the kinds of Māori engagement that has taken place, considerations for mātauranga Māori (traditional knowledge), benefit sharing, and intellectual property, as well as clear descriptions of how access or use of the information will benefit Kākāpō conservation (Dussex et al. 2021). This instance shows an emerging shift in tensions from extractive practice to cogovernance models that successfully meet the needs of Indigenous peoples as well as their research partners. Other similar international cases of note where these tensions have not been as successfully balanced include the San peoples and Hoodia, and the Kakadu Plum and First Nations Australians (Handsley-Davis et al. 2023, Brooks et al. 2024, Riddle et al. 2024b).

## Indigenous perspectives on benefit sharing

The issue of benefit sharing has become increasingly urgent considering historical and ongoing exploitation of Indigenous knowledge, resources, and land. Indigenous perspectives on benefit sharing are informed by values of reciprocity, intergenerational equity, community benefit, and self-determination (Villani et al. 2023). Indigenous peoples and their lands, waters, and territories are not merely sources of genetic materials or traditional knowledge, they are rights holders, who must be involved in decision-making and governance processes that affect their resources and futures. Benefit sharing, therefore, is not merely about distributing financial or technological returns. It is fundamentally about recognition, participation, and justice (Villani et al. 2023).

One of the critical concerns raised by Indigenous communities is the extractive model of science and biotechnology, which often involves taking genetic resources or knowledge without meaningful involvement or consent. In many cases, these resources are often used to develop profitable innovations, such as medicines or commercial products, without any return to the source communities (Handsley-Davis et al. 2023). While commercial entities may argue that the extensive cost of research and development for any one successful patent is already a significant investment with potential for “greater good” outcomes, this still violates the ethical principle of reciprocity and reinforces colonial patterns of exploitation, especially where there is potential for commercial profits. Benefit sharing should encompass a range of possible benefits, including:

Recognition of cultural authority and involvement in decision-making.
Participation in research design and implementation.
Recognition and attribution of traditional knowledge and custodianship.
Return of research findings in accessible and culturally appropriate formats.
Capacity-building for Indigenous researchers and community members.
Economic opportunities and revenue sharing from commercial outcomes.
Protection of cultural and spiritual values associated with biological resources.

Benefit-sharing can generally be categorized into two key types: monetary and nonmonetary. Each type offers distinct mechanisms for returning value to Indigenous communities, who contribute genetic resources or traditional knowledge. A summary of these types of benefit sharing can be found in Table 2.

**Table 2:** Types of benefit sharing.

Benefit type	Mechanism	Description	Example
Monetary	Royalties	A share of revenue or profits from products developed using Indigenous genetic resources.	Variant Bio pledged to share 4% of its revenue and 4% of its equity value with communities contributing DNA and health information (Variant Bio 2023a).
	Milestone payments	One-off financial payments made when specific stages of product development are achieved.	Payment upon successful commercialization or market entry.
	Research funding	Direct financial support for research conducted by, or in collaboration with, Indigenous communities	Funding Indigenous-led genetic or ecological research projects.
	Licensing fees	Fees paid for the right to use genetic resources or traditional knowledge in commercial or research contexts.	Licensing agreements for plant genetic material or associated traditional knowledge.
	Joint ventures	Business partnerships in which Indigenous communities share ownership, decision-making, risks, and profits with commercial entities.	Coowned biotech or research enterprises
	Trust funds	Dedicated funds set up to support conservation, cultural revitalization, education, or community development priorities, as identified by the Indigenous community.	Community-controlled funds for long-term development outcomes.
Nonmonetary	Capacity building	Training, knowledge exchange, and institutional development that support Indigenous-led research and innovation.	Training in synthetic biology, lab infrastructure development, or support for Indigenous research leadership (Schroeder et al. 2018).
	Technology transfer	The provision of tools, methodologies, and scientific equipment to support community-led initiatives and long-term research independence.	Transfer of scientific equipment into community research centers.
	Equitable access to medicines	Ensuring communities have free or affordable access to any therapeutics developed using their genetic resources.	Variant Bio has committed to making treatments affordable for partner communities, whose data first led to their discovery (Variant Bio 2023a).
	Collaborative research	Inclusion of Indigenous researchers and knowledge holders as equal partners.	Coauthorship of publications; participation in project governance.
	Education and training	Scholarships, internships, and tailored educational programs to build long-term community expertise.	Indigenous scholarships in science, law, or biotechnology.
	Infrastructure development	Building or upgrading schools, clinics, or research centers as a direct result of benefit-sharing agreements.	Schools, clinics, or research centers.
	Recognition of Indigenous rights	Formal legal acknowledgment of Indigenous ownership and governance over genetic resources and traditional knowledge.	Intellectual property arrangements and customary law recognition.

## Applying indigenous frameworks; key issues in relation to microorganisms

Several frameworks and guidelines set out the key issues that should be addressed for ethical engagement with Indigenous communities including benefit sharing. The following documents were chosen based on their relevance to Indigenous perspectives and/or applicability to microorganisms and reviewed to identify key issues relevant to ethical engagement and benefit sharing in biodiscovery research with microorganisms.

Rutzolijirisaxik Voluntary Guidelines for the Repatriation of traditional knowledge of Indigenous Peoples and Local Communities Relevant for the Conservation and Sustainable Use of Biological Diversity 2019.
Te Nohonga Kaitiaki Guidelines for Genomic Research with Taonga Species 2021.
He Tohu Arahi Guidelines 2024.
WAI262 Report—Ko Aotearoa Tenei 2011.
Te Rauika Mangai Guide for Wai262 2022.
Fungi Foundation Ethnomycology Ethical Guide 2023.
Queensland Biodiscovery—Traditional Knowledge Code of Practice 2021.

### Recognition of relational ontologies and taonga status in the Aotearoa New Zealand context

A fundamental issue within Aotearoa New Zealand is the recognition that microorganisms, like all life forms, possess mauri and whakapapa (genealogy) that link them to the broader ecological and spiritual systems of traditional knowledge. Te Nohonga Kaitiaki Guidelines stresses that taonga species are not only biological entities but also kin with whom Māori have reciprocal responsibilities (Hudson et al 2021). This means that microorganisms associated with taonga species or environments are not simply genetic resources, they are part of a living relational network that must be protected, maintained, and engaged with respectfully. Similarly, WAI262 acknowledges that the scope of Māori interest extends to all aspects of biodiversity, including those invisible to the naked eye, such as microorganisms (Waitangi Tribunal 2011).

### Governance and decision-making authority

Questions of governance, such as who decides how microorganisms are accessed, used, and shared, lie at the heart of equitable benefit sharing. Te Nohonga Kaitiaki outlines the importance of project-level, organizational, and systemic engagement with Māori as decision-makers, not just stakeholders (Hudson et al. 2021). He Tohu Ārahi reinforces this by asserting tino rangatiratanga over intellectual property, cultural knowledge, and research processes is key (Riddle et al. 2024b). While a shift toward Māori decision-making is crucial, there are practical challenges to address in terms of which Māori entities or collectives should exercise that responsibility on behalf of the relevant kaitiaki and beneficial communities. Ko Aotearoa Tēnei recommends the establishment of Māori advisory bodies and registers of kaitiaki interests to ensure Māori governance of taonga species, including microorganisms (Waitangi Tribunal 2011). Many intellectual property laws in Aotearoa New Zealand, such as the Plant Variety Rights Act (2022) and the Gene Technology Bill 110–1 (2024) have established Māori advisory groups to assess the role of kaitiaki in relation to their activities. These documents reflect a consistent demand for structural mechanisms that support Indigenous authority and control over microbial research and benefit sharing.

### Informed consent and ethical engagement

Meaningful benefit sharing cannot occur without the ongoing free, prior, and informed consent from those who have the authority to provide it (Department of Environment and Science 2021). This principle is embedded across all documents. Te Nohonga Kaitiaki advises that consent is a sustained relationship, characterized by mutual understanding and regular engagement. He Tohu Ārahi (2024) complements this by recommending detailed contracting processes and the use of tools such as the Local Contexts Traditional Knowledge and Biocultural Labels to communicate conditions of use, access restrictions, and expectations around sharing microbial data. This reflects the Labels categories of provenance, protocols and permissions. WAI262’s emphasis on consultation before the use of any biological materials underscores the same principle, that Indigenous rights must be actively considered at the earliest stages of research planning (2011). These recommendations are consistent with international best practice and the free, prior, and informed consent provisions found within the Nagoya Protocol and the UN Declaration on Rights of Indigenous Peoples (2007). It should be noted that informed consent and Indigenous governance should both be set up to provide the most robust and transparent approach to ethical engagement.

### Data sovereignty and digital sequence information

The rise of digital sequencing technologies has become a central concern in benefit sharing debates. Once microorganisms are sequenced, their digital genetic codes can be used, stored, offshored, and commercialized without any further material contact with the communities that consented the original biological source material. This risks bypassing Indigenous communities’ ability to establish mutually agreed terms for secondary uses of digital sequence information.

As a result, Indigenous data governance has emerged as a key discussion point in international policy discussions, including within the CBD and the Nagoya Protocol. Frameworks like He Tohu Ārahi and Te Nohonga Kaitiaki anticipate these challenges, calling for robust data management plans, the use of clear provenance and attribution protocols, and governance mechanisms that support Māori and Indigenous decision-making for both samples and data outputs (Maui Hudson et al. 2021, Riddle et al. 2024b).

Recent research undertaken at the University of Waikato reinforces the argument that digital sequence information has real and measurable economic value. In a scenario-based survey, researchers allocating a hypothetical research budget indicated an average willingness to pay 2.5% of their funds for access to digital sequence information (Coltman et al. 2025). This willingness increased significantly when data included clear provenance metadata and demonstrated Indigenous consent. These findings support the case for governance frameworks that embed transparency, accountability, and Indigenous participation, not just as ethical imperatives, but also as mechanisms that increase scientific legitimacy and uptake. Golan et al. (2022) argue that connecting digital sequence information to accurate and inclusive provenance metadata, specifically through systems like the Local Contexts Traditional Knowledge and Biocultural Labels and Notices, are vital for protecting Indigenous interests and creating pathways for future engagement and benefit sharing. Provenance information contextualizes data, enhances scientific reproducibility, and makes the ethical reuse of materials possible (Anderson et al. 2024). The Local Contexts Labels function as metadata tools that, with permanent IDs and project identifiers, can travel with data through repositories, signaling to researchers and institutions the existence of Indigenous interests, associated permissions, and culturally appropriate protocols for use.

Proper attribution, linking genetic material and digital sequence information to specific Indigenous communities, is not only a matter of ethical data practice, but essential for realizing fair and equitable benefit sharing (Raposo et al. 2024). Inclusive provenance metadata supports alignment with both the FAIR (findable, accessible, interoperable, and reusable) and CARE (collective benefit, authority to control, responsibility, and ethics) Principles, ensuring that Indigenous communities are recognized as costewards of resources and data rather than passive subjects (O’Brien et al. 2024, Sterner and Elliott 2024).

The use of Local Contexts’ Traditional Knowledge and Biocultural Labels is gaining international traction, with institutions like Manaaki Whenua (now the New Zealand Bioeconomy Science Institute) and the Smithsonian Institution now implementing them across large biological collections (Labels and Notices in Manaaki Whenua—Local Contexts 2022, Arenas et al. 2025). These Labels are interoperable across digital infrastructures and can be embedded into genomic data submissions, enabling Indigenous communities to define terms of access, attribution, and future use. They are especially useful in the absence of binding legal frameworks, but can also be used alongside declared public domain data sets. Such is their utility, that the Global Biodiversity Information Facility is working on four pilot integrations (with Ecuador, French Polynesia, Aotearoa, and USA) to see community and in-country use of the Local Contexts Labels recording community provenance, protocol, and permission information continue through into global repositories (Local Contexts 2025).

Together, these tools and findings illustrate that ethical and effective benefit sharing in the context of digital sequence information must go beyond monetary compensation. Provenance metadata, in particular is a prerequisite for data quality, future collaboration, and scientific integrity (Scholz et al. 2022, Raposo et al. 2024). This is particularly important in the context of microorganisms, where provenance can be harder to attribute and more likely to be strategically ignored. Recognizing Indigenous connections through robust data governance ensures that microorganisms remain connected to the communities and ecologies from which they originate.

### Commercialization and benefit sharing mechanisms

The economic value derived from microorganisms, whether through pharmaceuticals, agriculture, food technologies, or synthetic biology, raises the issue of how benefits can be defined and distributed to properly account for Indigenous rights and interests. Many frameworks insist that Indigenous communities receive a fair share of any financial gains and be involved in determining what meaningful benefits will be received through mutually agreed terms (Department of Environment and Science 2021). Te Nohonga Kaitiaki outlines mechanisms for capacity-building, coownership of intellectual property, and long-term investment in community priorities (Maui Hudson et al. 2021). He Tohu Ārahi advocates for benefit sharing to also include access to research results, educational opportunities, and infrastructure development (Riddle et al. 2024a). WAI262 warns against unilateral state or corporate decision-making that excludes Māori from benefit flows and emphasizes the moral imperative of returning benefits to source communities (Waitangi Tribunal 2011).

### Preventing misuse and misappropriation

The risk of biopiracy, where microorganisms or associated traditional knowledge are exploited without acknowledgment or benefit to source communities, is a long-standing concern. He Tohu Ārahi provides guidance on how to prevent such misuse through legal agreements, confidentiality clauses, and community coauthorship (Riddle et al. 2024a). Te Nohonga Kaitiaki encourages transparency and early dialogue to mitigate misunderstandings and unintentional harms (Hudson et al. 2021). WAI262 notes historical examples of exclusion and calls for systemic safeguards to prevent the recurrence of such patterns (Waitangi Tribunal 2011). These protections are crucial for microbial research, where exploitation can occur invisibly through data transfer and digital tools. (Potter and Māngai 2022). Potential pathways for addressing these issues are outlined within Table 3.

**Table 3:** Summary of implementation challenges and solutions.

Challenge	Description	Potential approaches; *direct* and indirect
Power imbalances	Significant disparities in negotiating power, legal literacy, and technical capacity between Indigenous communities and commercial or academic users.	*Capacity building to support Indigenous-led training initiatives, independent legal and advisory support*.
	Risk of exploitative or tokenistic agreements that do not reflect community priorities or rights.	Creation of example agreements, case studies, and community protocols as well as clear engagement protocols. *Independent legal advice and in-depth contracting discussions*.
Unclear ownership and entitlement	Authority to grant access and uncertainty over who holds authority to grant access to genetic resources at national and community levels.	Connect with and strengthen existing representative systems and groups. *Engage in good faith with both government representatives and local iwi*.
	Authority to distribute in a fair and equitable manner across differing beneficiary groups in accordance with their rights and responsibilities.	Advocacy for legal reform, leverage international law such as CBD. *Use of contracts to recognize customary law, and use of Local Contexts*.
	Lack of recognition for customary law or Indigenous jurisdiction in many national legal systems.	*Use biocultural protocols developed by the community*.
Ambiguity around the role of traditional knowledge	Difficulty determining whether benefit sharing should only apply when traditional knowledge is directly linked to the resource.	Use Indigenous guidelines, support Indigenous advocacy for rights recognition.
	Tension between traditional knowledge-based claims and broader claims grounded in custodianship of land, waters, and relational responsibilities.	Acknowledge different rights and responsibilities for traditional knowledge and genetic resources.
Cross-border and collective rights complexity	Species distribution may cover multiple communities and regions.	Coordinate with and support existing regional bodies, and community collectives such as Tribal fora.
	Challenges in distributing benefits fairly among multiple communities or across regions where a resource is found.	Develop protocols and guidelines based on existing case studies. *Utilization of both bilateral and multilateral benefit sharing mechanisms, with ability to expand to other communities directly in future*.
	Limited frameworks for recognizing collective rights at the international level.	Advocate for inclusion for and consideration of collective rights in international fora. *Use of contracts and mutually agreed terms*.
Weak or inconsistent legal frameworks	Variation in national access and benefit sharing laws and lack of binding international standards for nonstate actors.	Advocacy for domestic and international legal reform, leverage international law such as CBD. Establish a national benefit sharing authority to facilitate relationship building and connect parties.
	Enforcement mechanisms are often weak or absent.	Advocacy for legal reform, leverage international law such as CBD, *use of contracts to recognize customary law, and use of Local Contexts*.
Valuation and benefit design issues	Challenges in assigning fair value to genetic resources or associated traditional knowledge, especially before commercial potential is realized.	Promote and develop holistic valuation frameworks, rather than utility or downstream value alone. Recognize that different understandings of fair value vary between communities and can change over time. *Early conversations during research and creation of mutually agreed terms that recognize cultural, environmental, and commercial values*.
	Tension between monetary and nonmonetary benefits, and who decides what is meaningful.	*Codesign benefit sharing plans that cover both types of benefit sharing, and provide social, cultural, and environmental benefits*.

Note: italicized terms denote direct actions, and plain text indicates indirect actions.

### International landscape

In addition to these frameworks, benefit sharing mechanisms for Indigenous microorganisms are grounded in several international agreements that recognize the need to protect traditional knowledge and Indigenous rights and interests and provide normative principles and technical guidance for equitable distribution of benefits arising from genetic resource utilization (WIPO 2024, art. 8j). The CBD, adopted in 1992, articulates the foundational principles of sovereignty over genetic resources and the requirement that access to such resources is subject to prior informed consent and mutually agreed terms (Article 15). The CBD acknowledges traditional knowledge associated with biodiversity and sets expectations for its respectful use and equitable benefit sharing, particularly under Article 8(j) (CBD 1992).

To operationalize these commitments, the Nagoya Protocol was adopted in 2010. It codifies obligations for users of genetic resources to secure prior, informed consent from provider countries and Indigenous communities, and to negotiate mutually agreed terms for benefit sharing (CBD 2011). These benefits may be monetary, such as royalties, milestone payments, licensing fees, and research funding, or nonmonetary, including data sharing, collaborative research, technology transfer, education, training, and capacity building. This holistic view of benefit sharing is essential to ensuring that value circulates back to the communities from whom microbial and other genetic resources originate, and traditional knowledge is repatriated. The Rutzolijirisaxik Voluntary Guidelines, created by the CBD, stipulate the return of knowledge from whence it came to facilitate the recovery of traditional knowledge, without limiting its ongoing access and use unless under mutually agreed terms (Secretariat of the Convention on Biological Diversity 2019).

The recently established 2024 Cali Fund further expands the application of benefit sharing to digital sequence information, acknowledging that genetic resources are increasingly accessed digitally rather than through physical samples (CBD 2024). Under this new mechanism, companies using digital sequence information from genetic resources are expected to contribute 1% of net profits or 0.1% of revenue to the Cali Fund. These contributions will support global biodiversity conservation and deliver direct benefits to Indigenous communities, whose territories and knowledge systems actively conserve biodiversity. The Cali Fund is one of the first global efforts to address benefit sharing in the context of digital sequence information, and while participation is currently voluntary, its establishment at the CBD’s 16th Conference of the Parties underscores its significance.

WIPO has also contributed significantly to international discussions on the protection of traditional knowledge, including knowledge associated with microorganisms, through its Intergovernmental Committee on Intellectual Property and Genetic Resources, Traditional Knowledge and Folklore Intergovernmental Committee. While it was one of the reasons WIPO began its Intergovernmental Committee in 2001 (Anderson 2009), it has drafted articles that propose key protections for Indigenous knowledge systems. These include the recognition of collective Indigenous ownership, requirements for prior, informed consent, and provisions for fair and equitable benefit sharing (WIPO 2024). Although the draft articles remain under country negotiation and are not yet legally binding within nation states, they represent a meaningful step toward integrating Indigenous perspectives into international intellectual property systems and ensuring the respectful treatment of microbial knowledge.

A landmark shift also occurred with the adoption of the WIPO Treaty on Intellectual Property, Genetic Resources and Associated Traditional Knowledge (GRATK Treaty) in 2024. This treaty now mandates disclosure in patent applications regarding the origin of genetic resources, including microorganisms, and associated traditional knowledge (WIPO 2024). It further permits the development of searchable traditional knowledge databases to support patent examination and includes enforcement mechanisms, such as patent revocation for nondisclosure (WIPO 2024). While ground-breaking, its success will depend on national-level implementation and its integration with broader benefit sharing frameworks.

In summary, the convergence of Indigenous-led guidelines (e.g. Te Nohonga Kaitiaki, He Tohu Ārahi, and WAI262) and evolving international legal instruments (e.g. the GRATK Treaty, CBD, and Nagoya Protocol) points a clear direction for increasing Indigenous participation in the governance of genetic resources. These instruments collectively reveal that the core issues around access and benefit sharing apply equally to microorganisms and extend beyond narrow legal compliance to restoring balance in relationships between peoples, species, and knowledge systems. These sources call for a profound rethinking of how science is done, how benefits are defined, and how Indigenous sovereignty can be upheld in an age of rapid technological advancement. As such, they offer both ethical direction and practical tools for researchers, institutions, and policymakers engaging with microbial genetic resources sourced from Indigenous territories.

Implementing benefit sharing in practice, particularly in the context of Indigenous microorganisms and genetic resources, presents a range of complex challenges. One of the most persistent issues is the power imbalance between Indigenous communities and researchers or commercial entities. Disparities in negotiating power, technical expertise, and financial resources can lead to inequitable agreements that fail to reflect the contributions or rights of Indigenous peoples. Similarly, a lack of transparency about the commercialization process, the costs and returns, can create unrealistic expectations about what constitutes a fair and equitable return. In many jurisdictions, legal frameworks governing access and benefit sharing remain underdeveloped or inconsistently applied, meaning that only a fraction of users contribute monetary benefits (Sara et al. 2022). Legal uncertainty creates risks for both providers and users of genetic resources and undermines confidence in access and benefit sharing arrangements.

Distributing benefits fairly and equitably among different communities can be a major challenge, especially when research spans multiple regions, Indigenous groups, or even countries. Determining how to allocate benefits in such cases can be politically and ethically fraught. Ensuring long-term compliance with benefit sharing commitments also remains difficult, particularly where digital sequence information is used and the physical connection to original genetic material is obscured. Moreover, assigning economic value to genetic resources or associated traditional knowledge is a complex and often speculative exercise, especially in early stages of research when commercial potential is uncertain.

To address these challenges, several best practices and solutions have emerged. First, the development and use of community protocols can help center Indigenous values, priorities, and decision-making processes in access and benefit sharing negotiations. These protocols provide guidance on how communities wish to be approached, how decisions should be made, and what constitutes fair engagement.

Applying the principle of free, prior, and informed consent is also essential, not as a one-off checkbox, but as a continuous process that respects the right of communities to decline, change their minds, or renegotiate terms as projects evolve. It is also important to note however that many communities themselves are most likely able to navigate this complexity through their own decision-making processes and relational frameworks.

Multilateral agreements are also one of the most useful tools for navigating the balance of multiple community interests, but the size and scope of these mechanisms need careful attention. For example, the CBD Digital Sequence Information Cali fund is a global fund that is useful for benefit sharing in a situation, where multiple countries' data is used, or the Brazil National Fund meets a national level benefit sharing need (Brooks et al. 2024). In some cases, it may be apropriate to set up bespoke benefit sharing funds or arrangements for specific species and community contexts (Brooks et al. 2024). Different levels, such as national and global levels, are not always appropriate scales for use cases and there is also a need for more flexible subnational processes for agreement making. Developing these entities for access and benefit sharing, or processes for their establishment, would be useful.

Building the capacity of Indigenous communities is a key strategy for addressing power imbalances. Legal training, technical education, and negotiation skills development can help communities engage more confidently and effectively in benefit sharing arrangements. Using standardized access and benefit sharing templates can streamline the negotiation process and reduce the pressure on communities to draft agreements from scratch. The involvement of independent third parties, such as government agencies, nongovernmental agencies, legal experts, or community advocates, can provide additional oversight and ensure that agreements are transparent and fair.

Creating registries or databases to track the use of genetic resources and associated digital sequence information can help monitor compliance and uphold access and benefit sharing obligations. These tools support transparency and can be designed to ensure community access and control over data.

Finally, maintaining ongoing engagement with communities throughout a research or commercialization project is crucial. Regular consultations help identify shifting needs and priorities and ensure that benefit sharing continues to reflect what is most valuable to the community.

One of the most effective tools to create legal certainty are agreements and contracts (Anderson et al. 2024). While varying legal systems and jurisdictions may uphold Indigenous rights and interests differently, many are limited in scope and enforceability. The creation of agreements and contracts between Indigenous peoples and those seeking to engage with them is the most efficient, and legally enforceable way that Indigenous peoples can ensure that parameters and protections are in place. These contracts should include mutually agreed terms, benefit sharing arrangements, data protection, and intellectual property plans. Independent legal representation for Indigenous communities throughout the contracting process is essential for ensuring equitable negotiation outcomes. There are multiple ways that benefit sharing mechanisms can be set up through this contract; a bilateral agreement directly between the contracting parties, or a collective bilateral agreement between contracting parties where multiple communities may be involved (Whare 2021).

There is also the third-party environment, where a multilateral benefit sharing arrangement occurs, such as the Cali Fund. A hybrid solution may also be used, where contracting parties engage in both direct, bilateral benefit sharing, as well as a third-party benefit sharing mechanism. In situations where there are too many uncertainties remaining unsolved, those who will be sharing benefits are more likely to use third party benefit sharing mechanisms to meet their need for legal certainty and license to operate.

## Emerging models and innovations

The integration of Indigenous data sovereignty and governance into benefit sharing frameworks is an emerging best practice, affirming Indigenous peoples’ rights to control how data about their resources, lands, and people is collected, used, and shared.

The Global Indigenous Data Alliance developed the CARE Principles, which provide ethical guidance for data use and help shape culturally appropriate benefit sharing models (Carroll et al. 2020). The CARE principles, as well as the FAIR Principles and the TRUST Principles (transparency, responsibility, user-focus, sustainability, and technology) have been referred to in the recent CBD digital sequence information mechanism, where entities operating public databases, tools, and models that are dependent on digital sequence information should operate in a manner consistent with them (decision 16/2 CBD 2024; CBD 2024). As this mechanism is still relatively new, the practical operationalization of these principles within public databases is still maturing.

A particularly useful emergent innovation in this space is the growing use of Local Contexts’ Traditional Knowledge and Biocultural Labels. These Labels allow Indigenous communities to digitally mark data, research outputs, biological materials, and a myriad of other items with culturally relevant metadata that communicates a community’s expectations around provenance, protocols and permissions for use. For example, a Traditional Knowledge Label might specify that certain knowledge is sacred, not for commercialization, or requires ongoing consultation (Local Contexts 2022). Biocultural Labels can similarly indicate the governance protocols associated with genetic resources, such as microorganisms, and provide visibility and recognition in scientific databases where such context is typically absent. When integrated into data platforms, public databases, or research agreements, the Labels function as a practical expression of Indigenous data sovereignty and a tool for safeguarding ethical engagement in research. Their use helps to operationalize FAIR and CARE Principles, as well as aligns data with emerging data governance protocols that support community governance and accountability in benefit sharing practices (Anderson et al. 2024).

A summary of implementation challenges and emergent solution approaches can be found below in Table 3.

## Case studies in benefit sharing

The following case studies are based on contributions provided directly by collaborators and are presented here in descriptive form to reflect their input.

### Basecamp research: ethical biodiscovery and access and benefit sharing

Basecamp Research is a UK-based biotech company specializing in AI-driven protein design (Basecamp Research 2024). It has developed one of the largest global protein databases by sequencing environmental samples collected through access and benefit sharing partnerships in over 25 countries (Basecamp Research 2024). Environmental samples frequently contain diverse microbial communities, meaning that microorganisms and their genetic material may also be collected and analysed during biodiversity research initiatives. The company’s access and benefit sharing approach is built on the principles of the CBD and the Nagoya Protocol. Before collecting samples or accessing genetic resources, Basecamp Research establishes formal, mutually beneficial ABS agreements with governments, research institutions, and local communities. The agreements comply with national legislation, as well as the obligations of the CBD and the Nagoya Protocol, ensuring transparent and equitable access and benefit sharing from the utilization of genetic resources. Notably, Basecamp recognizes the importance of benefit sharing from digital sequence information even in jurisdictions, where regulatory frameworks for digital sequence information are not yet established or fall under international jurisdiction, such as international waters.

In 2024, Basecamp signed a historic access and benefit sharing agreement with the Government of Cameroon, marking the country’s first formal agreement covering digital sequence information (Kasianov 2024). Key features included:

Community engagement: four communities in Cameroon consented to sample collection after a consultation process.
Royalty framework: the company committed to paying royalties when Cameroonian digital sequence information is used in developing any commercial applications. Royalty agreements additionally cover the use of data in training precedent-setting AI foundation models.
Capacity building: basecamp supports local scientists through training, lab development, data access, and research funding.
Local partnerships: as a pilot, the company partnered with AJESH, a Cameroonian nonprofit focused on biodiversity conservation and climate adaptation. This collaboration has since been expanded to work with scientists at the University of Buea.
Recognition of DSI: the agreement explicitly values digital sequence information and ensures financial benefits are equitably shared.
Efforts being made from Basecamp may lead to stronger collaborative relationships, which can create long-term benefit for the community.

### Variant Bio: community driven benefit sharing in genomics

Variant Bio uses human genomic diversity to discover and develop new therapies, with a model based on deep respect for community partners (Variant Bio 2023b). It prioritizes equity, transparency, and long-term relationships, particularly in the context of development of disease treatments (Variant Bio 2023a). Research on human genetic diversity may also include analysis of human-associated microbiomes, meaning that microbial genetic resources connected to Indigenous peoples could also be accessed and utilized, raining similar issues of consent, governance, and benefit sharing.

The company’s benefit sharing program includes both immediate and future financial and material benefits, in addition to other important benefits such as scientific capacity building, data cogeneration with local study partners, and the sharing of relevant health information and research results with participants (LeBaron von Baeyer 2024a):

Short-term: 10% of every project’s study budget (up to a maximum of $100 000 USD) in support of health, education, environment, and cultural needs based on community consultations.
Long-term: A 4% share of revenue on an annual calendar basis and 4% equity value (should the company be acquired or go public) is pledged to be divided equally among contributing communities (Variant Bio 2023a).

### Affordable medicine pledge

Commitments to provide any resulting treatments or tests at an affordable price to partner communities whose data first led to their discovery. In collaboration with the University of Antananarivo, Variant Bio launched a genetic research project in Madagascar, structured around a highly participatory engagement model (Rakotoarivony 2024). Key Features included:

Initial engagement (December 2020–February 2021): meetings with community leaders and residents across three regions.
Community needs assessment: identification of common benefit sharing priorities such as clean water, sanitation, and school infrastructure.
Voting process: during sample collection, 257 participants filled out an exit survey on benefit sharing priorities.

Implemented initiatives:

Rebuilding a village water pump in Tsianaloka Village on the West Coast.

Roofing materials for two schools in Tsiandatsiana Village and Ampandrialaza Village in the Southern and Central Highlands.

Lab equipment and training for students at the University of Antananarivo.

Innovative features:

Community-driven decision-making.

Integration of ethics and free, prior, and informed consent.

Transparency and public documentation.

Strengthened scientific capacity through infrastructure and training (LeBaron von Baeyer 2024b).

This model demonstrates how codesigned benefit sharing can foster trust, deliver meaningful benefits, and support long-term development.

### Fungi Foundation: ethical ethnomycology and indigenous engagement

The Fungi Foundation (FFungi) is a global nonprofit with the purpose of exploring, documenting, and conserving fungi, with a strong emphasis on traditional knowledge and partnerships. Part of their approach includes the launch of the Elders Program, which partners with Indigenous communities to document their ancestral relationships with fungi. FFungi have also published Ethnomycology Ethical Guidelines in 2023, providing a living framework to ensure ethical conduct, reciprocal relationships, and respect for Indigenous perspectives and traditions in relation to fungi (Villani et al. 2023). Key features included:

Traditional knowledge documentation: Catalogd over 200 traditional uses for fungi across 45 countries and 98 distinct Indigenous or local groups.
Ethical framework: The 2023 Ethical Guidelines center Indigenous worldviews and perspectives, encourage meaningful ongoing relationships, and assert that biodiversity conservation and appropriate knowledge transmission are inextricably linked.
Education and conservation: FFungi advocate for the integration of fungal knowledge in schools and environmental policy, as it is essential for the elevation of fungi into similar importance as flora and fauna.

### Capacity building and benefit sharing

Community leadership: Indigenous communities guide how fungal knowledge is recorded and shared, with FFungi ensuring coauthorship, informed consent, and shared stewardship of data.

### Cultural and environmental sustainability

Through incorporating traditional knowledge and elevating fungi in environmental discourses, the foundation promotes the safeguarding of Indigenous cultural intellectual property and biodiversity.

### Innovative elements

Equity in Ethnomycology: contributes to the elevation of Indigenous science and traditional knowledge alongside modern science to support biodiversity conservation and scientific advancement.

Legal status elevation: advocates for the integration of fungi into legally recognized components of ecosystems, as has been done in Chilean law.

Flexible governance model: the ethical guidelines emphasize the need to have protocols rooted in cultural principles and enable Indigenous communities to assert their priorities and governance in fungal research and knowledge management.

### Kichwa de Sarayaku territory case study

In a collaboration with More than Human Life (MOTH 2025a) program at NYU Law, and in support of the Kawsak Sacha (Living Forest) Declaration, recognizing the forest as a living entity, the Kichwa People of Sarayaku invited the Society for the Protection of Underground Networks (SPUN) and FFungi to sample and document the fungi diversity of the forest both above ground and within the soil (Arenas et al. 2025). The research team, alongside Sarayaku mycologists ran an educational awareness program in local schools to teach youth about the local fungi, and recorded ethnomycological oral histories of the community as well as musical knowledge transmission, promoting an increase in knowledge exchange and capacity building in the community (Fungi Foundation 2025). With time, it is likely that further benefits will be forthcoming.

FFungi offer a comprehensive model of knowledge management and benefit sharing. Their Elders Programme and Ethical Guidelines place Indigenous governance at the core of fungal research, showing how nonmonetary benefit sharing can empower communities as custodians of their own knowledge as well as of biodiversity.

Additionally, this project forms part of the Science Across Cultures Initiative (SACI), a collaboration between the Sarayaku Nation, the MOTH Program, SPUN, FFungi, Cosmo Sheldrake, and Local Contexts, which aims to bridge Indigenous and Western scientific epistemologies to support legal and political recognition of the living forest (Kawsak Sacha) (MOTH 2025b). As part of SACI, the team not only documented fungal diversity and soil fungal DNA sequencing but also used bioacoustic devices to record soundscapes of the forest and river to weave together multiple dimensions of understandings of the forest. To protect Sarayaku sovereignty, data generated through the project is governed under memorandums of understanding, as well as the use of the Local Contexts Labels (MOTH 2025b, Arenas et al. 2025). Significantly, the MOU names both the Sarayaku and the Kawsak Sacha as the stewards holding the primary cultural responsibilities for the information and data. The Sarayaku Labels mirror this explicit recognition of rights in the agreement which was signed by all parties on Sarayaku territory.

## Conclusion

Interest in biodiscovery research with microorganisms is increasing fueled by advances in genomics and artificial intelligence. Current international mechanisms, such as the CBD, the Nagoya Protocol, and the WIPO GRATK Treaty, have begun to respond to the complexities of access and benefit sharing in the age of digital sequence information. However, these frameworks often lag behind the technological realities of genomic science and fail to adequately reflect Indigenous worldviews. As a result, many Indigenous communities remain vulnerable to exclusion, misappropriation, and tokenistic engagement.

To ensure that benefit sharing is not merely transactional but transformative, it must be rooted in the principles of self-determination, reciprocity, and Indigenous data sovereignty. This requires a shift from extractive models of science toward cogovernance, codevelopment, and relational accountability. The emerging practices outlined, such as the community driven partnerships led by Variant Bio, Basecamp Research, and FFungi offer promising models for ethical engagement and partnership, but they must be scaled, institutionalized, and consistently evaluated through Indigenous-led mechanisms. We encourage researchers and institutions to build on these examples, using the elements that work in their contexts and creating other innovative approaches to ensuring Indigenous participation and benefit sharing are normalized as part of the scientific process.

The governance of microorganisms and the equitable sharing of benefits derived from their use represent a critical intersection between biotechnology, international law, and Indigenous rights. Microorganisms, particularly those connected to Indigenous lands, waters, and traditional knowledge, are relational beings embedded in Indigenous cosmologies, and their use raises ethical, cultural, and political considerations that are not yet fully integrated into conventional access and benefit sharing frameworks.

Ultimately, benefit sharing must not only address historical injustices but also build futures that uphold the mana, rights, and responsibilities of Indigenous peoples. This includes recognizing microorganisms as taonga, embedding Indigenous protocols in research design, ensuring full and ongoing free, prior, and informed consent, and sharing both monetary and nonmonetary benefits in ways defined by communities themselves. As international legal norms evolve and scientific frontiers expand, it is imperative that Indigenous leadership be centered, not as stakeholders, but as rights holders and sovereign partners in shaping the future of microbial research and innovation.
